# Determining the Case Fatality Rate of COVID-19 in Italy: Novel Epidemiological Study

**DOI:** 10.2196/32638

**Published:** 2022-02-10

**Authors:** Mengqing Yan, Wenjun Kang, Zhifeng Guo, Qi Wang, Peizhong Peter Wang, Yun Zhu, Yongli Yang, Wei Wang

**Affiliations:** 1 Department of Occupational and Environmental Health College of Public Health Zhengzhou University Zhengzhou China; 2 The Key Laboratory of Nanomedicine and Health Inspection of Zhengzhou Zhengzhou China; 3 Center for New Immigrant Wellbeing Markham, ON Canada; 4 Dalla Lana School of Public Health University of Toronto Toronto, ON Canada; 5 Department of Epidemiology and Biostatistics Tianjin Medical University Tianjin China; 6 Department of Epidemiology and Biostatistics College of Public Health Zhengzhou University Zhengzhou China

**Keywords:** COVID-19, case fatality rate, discharged case fatality rate, new infectious diseases

## Abstract

**Background:**

COVID-19, which emerged in December 2019, has spread rapidly around the world and has become a serious public health event endangering human life. With regard to COVID-19, there are still many unknowns, such as the exact case fatality rate (CFR).

**Objective:**

The main objective of this study was to explore the value of the discharged CFR (DCFR) to make more accurate forecasts of epidemic trends of COVID-19 in Italy.

**Methods:**

We retrieved the epidemiological data of COVID-19 in Italy published by the John Hopkins Coronavirus Resource Center. We then used the proportion of deaths to discharged cases（including deaths and recovered cases） to calculate the total DCFR (tDCFR), monthly DCFR (mDCFR), and stage DCFR (sDCFR). Furthermore, we analyzed the trend in the mDCFR between January and December 2020 using joinpoint regression analysis, used ArcGIS version 10.7 to visualize the spatial distribution of the epidemic CFR, and assigned different colors to each province based on the CFR or tDCFR.

**Results:**

We calculated the numbers and obtained the new indices of the tDCFR and mDCFR for calculating the fatality rate. The results showed that the tDCFR and mDCFR fluctuated greatly from January to May. They first showed a rapid increase followed by a rapid decline after reaching the peak. The map showed that the provinces with a high tDCFR were Emilia-Romagna, Puglia, and Lombardia. The change trend of the mDCFR over time was divided into the following 2 stages: the first stage (from January to May) and the second stage (from June to December). With regard to worldwide COVID-19 statistics, among 6 selected countries, the United States had the highest tDCFR (4.26%), while the tDCFR of the remaining countries was between 0.98% and 2.72%.

**Conclusions:**

We provide a new perspective for assessing the fatality of COVID-19 in Italy, which can use ever-changing data to calculate a more accurate CFR and scientifically predict the development trend of the epidemic.

## Introduction

COVID-19, which emerged in December 2019, has spread rapidly around the world and has become a serious public health event endangering human life [1,2]. The COVID-19 pandemic has strained or overwhelmed health systems across the world, with over 128 million COVID-19 cases and 2.8 million deaths as of March 31, 2021 [3]. Reports indicate that more than 200 countries have confirmed COVID-19 cases [4]. COVID-19 has high infection and mortality rates, and thus, it has become a pandemic [5,6]. Numerous studies have been conducted since the outbreak of COVID-19, and the reported findings have provided insights into the prevention and control of the disease [7,8]. With regard to COVID-19, there are still many unknowns, such as the exact case fatality rate (CFR) and the speed at which it spreads across communities. This lack of evidence complicates the design of appropriate response policies.

The European population was severely hit by the COVID-19 outbreak, particularly in Italy, which was the first European country to be affected by COVID-19 [9]. The first case of pneumonia due to SARS-CoV-2 in Italy, without a history of possible exposure abroad, was diagnosed in Lombardy (Northern Italy) on February 20, 2020. Within a few days, several COVID-19 cases were confirmed in the surrounding areas, and they included a substantial number of critically ill patients. Based on the number of cases and the advanced disease stage, it was estimated that community spread had been occurring since January 2020 [10]. Current statistics indicate that Italy is one of the countries severely affected by COVID-19–induced pneumonia [11]. By March 31, 2021, Italy had reported 3,584,899 confirmed COVID-19 cases and 109,346 deaths, ranking sixth worldwide in the number of deaths. Moreover, the CFR was approximately 3.05%, but it continues to fluctuate. A recent study analyzing over 70,000 COVID-19 patients in Italy revealed a wide variability in the CFR [12]. Therefore, the true incidence and CFR in Italy might be underestimated. There are some limitations in traditional mortality assessment methods. In order to solve these limitations and shortcomings, we introduce a new assessment method.

The purpose of our research was to determine how to make full use of the ever-changing authoritative data to make more accurate trend predictions of the epidemic. In order to more accurately assess the actual situation of the COVID-19 epidemic, we explored the use of the discharged CFR (DCFR) instead of the CFR to estimate the true situation and the use of the DCFR to make more accurate forecasts of epidemic trends. Public health institutions can use this method to calculate the dynamic fatality rate in different regions in real time, evaluate the medical conditions in different regions, and scientifically guide and reasonably arrange follow-up medical approaches. In addition, after entering the “turning point,” the overall situation of the entire epidemic can be predicted in advance based on the development data of the epidemic at that time and with reference to the real-time dynamic fatality rate data.

## Methods

### Data Collection and Characteristics

We obtained daily case reporting data from the Johns Hopkins University Center for Systems Science and Engineering (CSSE) [13]. The Center’s time-series data provided cumulative totals of COVID-19 cases and deaths by country [3]. The CSSE pools data from multiple sources, including the World Health Organization, the European Centre for Disease Prevention and Control, and the US Centers for Disease Control and Prevention, to produce daily country totals of confirmed cases and deaths. Data on COVID-19 in Italy involve daily updates from the Italian Ministry of Health managed by the Civil Protection Department.

### Statistical Analysis

#### Estimation of the DCFR

In this study, the DCFR and 95% CIs were estimated at the national and provincial levels of Italy. In addition, we selected several countries with a high number of confirmed cases, and the same method was performed to describe the death rate. ArcGIS software (version 10.7; Esri) was used to visualize the spatial distribution of the epidemic CFR and assign different colors to each province based on the CFR or total DCFR (tDCFR).

Notably, the DCFR includes the tDCFR, the monthly DCFR (mDCFR), and the stage DCFR (sDCFR). Discharged cases include deaths and recovered cases. The tDCFR is the proportion of deaths among discharged cases in the entire pandemic, the mDCFR is the proportion of deaths among discharged cases in each month, and the sDCFR is the proportion of deaths among discharged cases at each stage. The CFR, tDCFR, mDCFR, and sDCFR were calculated and analyzed as follows:

CFR = (number of deaths attributed to COVID-19 / number of total confirmed case of COVID-19) × 100% **(1)**

tDCFR = (number of total deaths attributed to COVID-19 / [number of total deaths attributed to COVID-19 + number of total recovered cases]) × 100% **(2)**

mDCFR = (number of monthly deaths attributed to COVID-19 / [number of monthly deaths attributed to COVID-19 + number of monthly recovered cases]) × 100% **(3)**

sDCFR = (number of total deaths at each stage attributed to COVID-19 / [number of total deaths at each stage attributed to COVID-19 + number of total recovered cases at each stage]) × 100% **(4)**

The CFR, tDCFR, mDCFR, and sDCFR were estimated with 95% CIs. CI is an interval range containing population parameters constructed under a certain degree of confidence, and is widely used to estimate the range of population parameters [14,15]. We calculated the 95% CIs through a normal approximate method. The following formula was used to calculate the CIs [16,17]:

95% CI = (*P* − *Z*_a/2_
*S_P_*, *P* + *Z*_a/2_
*S_P_*) **(5)**

where *P* = *n*/*N*, *Z*_a/2_ = 1.96 for a 95% CI*, and S_P_* *=
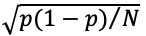
.*

#### Trend Analysis

Furthermore, we analyzed the trend in mDCFR between January and December 2020 using joinpoint regression analysis. Joinpoint analysis used the Joinpoint Regression Program (version 3.4.3). The joinpoint is a point in the trend curve where a statistically significant change in trend over time is observed. The analysis requires a minimum number of 3 observations from a joinpoint to either end of the data and a minimum number of 4 observations between 2 joinpoints. From the joinpoint regression model, we extracted the monthly percentage change (MPC) and the average monthly percentage change (AMPC). The MPC is calculated for each significant trend from a piecewise log-linear model on the logarithm of the age-standardized rate versus the year, while the AMPC represents the average of MPC estimates per significant trend weighted by the corresponding trend length (number of years in the trend). The trend analysis using the joinpoint regression model was performed by the SEER*Stat software [18] (Joinpoint Trend Analysis software from the Surveillance Research Program of the US National Cancer Institute; version 4.8.0.1 [19]).

## Results

### COVID-19 Situation in Italy

By December 31, 2020, in Italy, the incidence rate in 2020 was 3.49%, the annual CFR was 3.52%, and the mortality rate was 123.11 per 100,000 cases. The first confirmed case of COVID-19 in Italy was reported on January 31, 2020, and the numbers showed a slight increase each month after February. The first peak was observed in March, but the number decreased from April to a minimum in July. In August 2020, the number of newly diagnosed patients rose to 21,677, followed by a sharp rise in November to reach the second peak of 922,124 new cases. With regard to COVID-19–related deaths, the number was relatively small in February, but the number increased rapidly from March, exceeding 10,000 deaths per month. The highest number of deaths (15,549) was recorded in April. However, the number declined in the following months, with the lowest (374) being recorded in August. Moreover, the number of new deaths in September and October remained low, but the number rose sharply in November (Table 1).

**Table 1:** The characteristics of COVID-19 in Italy in 2020.

Month	Total confirmed cases, n	Monthly confirmed new cases, n	Total recovered, n	Monthly recovered, n	Total deaths, n	Monthly deaths, n	CFR^a^ (%), value (95% CI)	tDCFR^b^ (%), value (95% CI)	mDCFR^c^ (%), value (95% CI)
1	2	2	0	0	0	0	0	0	0
2	1128	1126	46	46	29	29	2.57 (−19.36 to 24.51)	38.67 (27.65 to 49.69)	38.67 (27.65 to 49.69)
3	105,792	104,664	15,729	15,683	12,428	12,399	11.75 (9.87 to 13.63)	44.14 (43.56 to 44.72)	44.15 (43.57 to 44.73)
4	205,463	99,671	75,945	60,216	27,967	15,539	13.61 (13.41 to 13.82)	26.91 (26.64 to 27.18)	20.51 (20.22 to 20.80)
5	232,997	27,534	157,507	81,562	33,415	5448	14.34 (14.19 to 14.49)	17.50 (17.33 to 17.67)	6.26 (6.10 to 6.42)
6	240,578	7581	190,248	32,741	34,767	1352	14.45 (14.31 to 14.59)	15.45 (15.30 to 15.60)	3.97 (3.76 to 4.17)
7	247,537	6959	199,974	9726	35,141	374	14.20 (14.06 to 14.34)	14.95 (14.8 to 15.09)	3.70 (3.33 to 4.07)
8	269,214	21,677	207,653	7679	35,483	342	13.18 (13.05 to 13.31)	14.59 (14.45 to 14.73)	4.26 (3.82 to 4.71)
9	314,861	45,647	227,704	20,051	35,894	411	11.40 (11.28 to 11.52)	13.62 (13.49 to 13.75)	2.01 (1.82 to 2.20)
10	679,430	364,569	289,426	61,722	38,618	2724	5.68 (5.60 to 5.76)	11.77 (11.66 to 11.88)	4.23 (4.07 to 4.38)
11	1,601,554	922,124	757,507	468,081	55,576	16,958	3.47 (3.43 to 3.51)	6.84 (6.78 to 6.89)	3.50 (3.44 to 3.55)
12	2,107,166	505,612	1,463,111	705,604	74,159	18,583	3.52 (3.49 to 3.55)	4.82 (4.79 to 4.86)	2.57 (2.53 to 2.60)

^a^CFR: case fatality rate.

^b^tDCFR: total discharged case fatality rate.

^c^mDCFR: monthly discharged case fatality rate.

### CFR, tDCFR, and mDCFR

Based on the daily report of COVID-19 cases in Italy, we used the new definition method described above to calculate the number of deaths and the number of recovered patients to obtain the new indices tDCFR and mDCFR for calculating the fatality rate. By December 31, 2020, the CFR in Italy was 3.52% and the tDCFR was 4.82% (Table 1).

**Figure 1:**
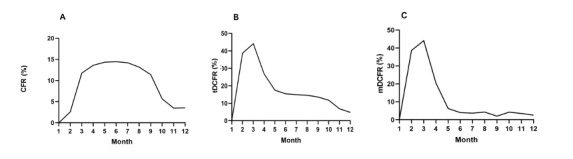
The trends of the (A) case fatality rate (CFR), (B) total discharged case fatality rate (tDCFR), and (C) monthly discharged case fatality rate (mDCFR) of COVID-19 in Italy.

Figure 1 shows the trends of the CFR, tDCFR, and mDCFR of COVID-19 in Italy from January 31, 2020, to December 31, 2020. The results indicated that the CFR first rose, then fell, and finally stabilized. The highest value of 14.45% was observed in June, and it stabilized at about 3% after November (Figure 1A). On the other hand, the tDCFR and mDCFR fluctuated greatly. The results indicated that the tDCFR and mDCFR fluctuated greatly from January to May. They first showed a rapid increase followed by a rapid decline after reaching the peak (Figure 1B). The highest values of the tDCFR and mDCFR were observed in March (44.14% and 44.15%, respectively). After May, the tDCFR gradually decreased and finally stabilized at about 4%, while the mDCFR first declined after May and then showed a slight upward trend in August, and after falling to the lowest value of 2.01% in September, there was a slight increase in October, but it finally stabilized at around 2.5%.

### Calculation of the CFR and tDCFR for 20 Provinces in Italy

**Figure 2:**
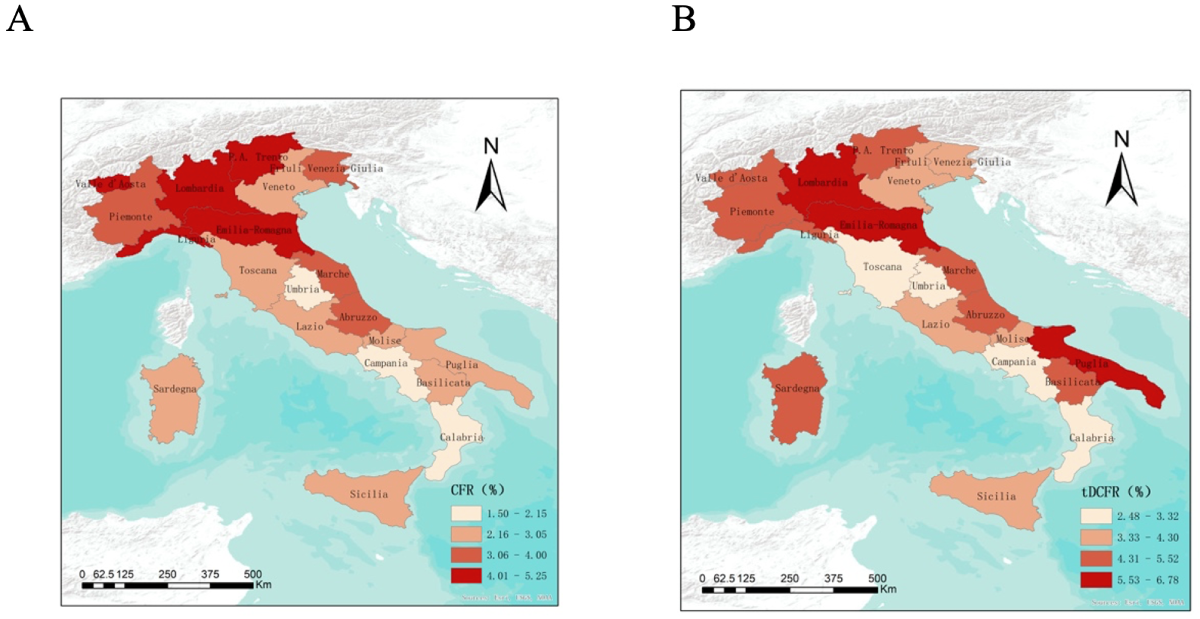
Map showing the (A) case fatality rate (CFR) and (B) total discharged case fatality rate (tDCFR) in Italian provinces.

The provinces are divided into 4 levels according to the value of the CFR or tDCFR, which are represented by 4 colors. Among them, red indicates areas with high CFR or tDCFR values, while white indicates areas with low values. The map shows that the provinces with high CFR values were Lombardia, Valle d'Aosta, Liguria, Emilia-Romagna, P.A. Trento, and Piemonte. In addition, there were 3 regions with high tDCFR values, including Emilia-Romagna, Puglia, and Lombardia. The results showed that the fatality rate of the epidemic, whether the CFR or tDCFR, was significantly higher in Northern Italy than in the Southern regions. However, there were some differences between the 2 evaluation indicators. For example, in the Puglia region, the tDCFR was 6.51% but the CFR was 2.72%, and in the Basilicata region, the tDCFR was 5.36% but the CFR was 2.36% (Figure 2).

### Estimation of Pandemic Stages From the Calculated mDCFR and sDCFR

**Figure 3:**
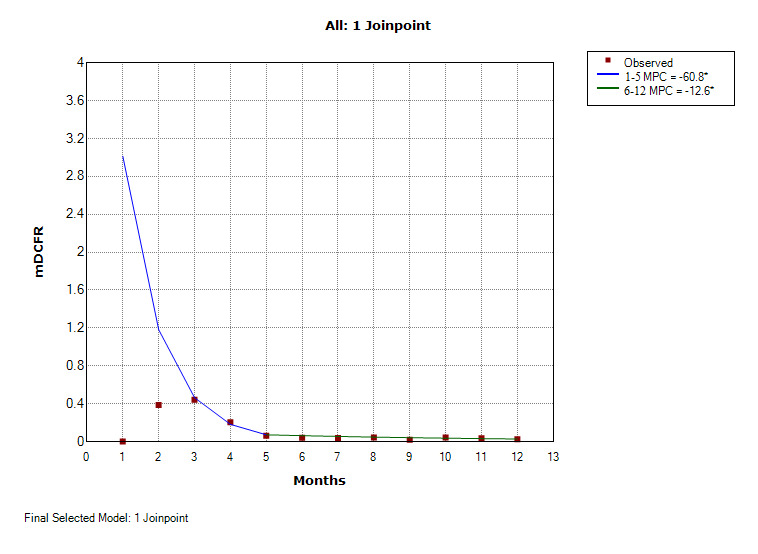
Estimation of pandemic stages in Italy using the monthly discharged case fatality rate (mDCFR). *The monthly percentage change (MPC) is significantly different from 0 at the alpha level of .05.

We used the Joinpoint Regression Program to analyze the change trend of the mDCFR over time, and determine a segmentation point, which was divided into 2 stages (Figure 3). The first stage was a sharp decline from January to May 2020, with the MPC being −60.8 (95% CI −69.6 to −49.5; *P*<.001). The second stage ranged from June to December 2020, with a sharp decrease in the declining stage, and the MPC was −12.6 (95% CI −21.0 to −3.5; *P*=.02). In comparison, the global AMPC for months 1 to 12 was −34.7 (95% CI −40.6 to −28.3; *P*<.001). The first phase represented the outbreak period, while the second phase represented the stable period. In addition, we calculated the sDCFR of each stage based on the number of deaths and the number of recovered cases in the different stages in Italy (Table 2). The results showed that the sDCFR values of the first and second stages were 17.50 (95% CI 17.33-17.67) and 3.03 (95% CI 3.00-3.06), respectively.

**Table 2:** The stage discharged case fatality rate of COVID-19 at different stages in Italy in 2020.

Stage	Time period	Deaths, n	Recovered cases, n	sDCFR^a^ (%), value (95% CI)
The first stage	January 31 to May 31	33,415	157,507	17.50 (17.33-17.67)
The second stage	June 1 to December 31	40,744	1,305,604	3.03 (3.00-3.06)

^a^sDCFR: stage discharged case fatality rate.

### Worldwide COVID-19 Statistics

**Figure 4:**
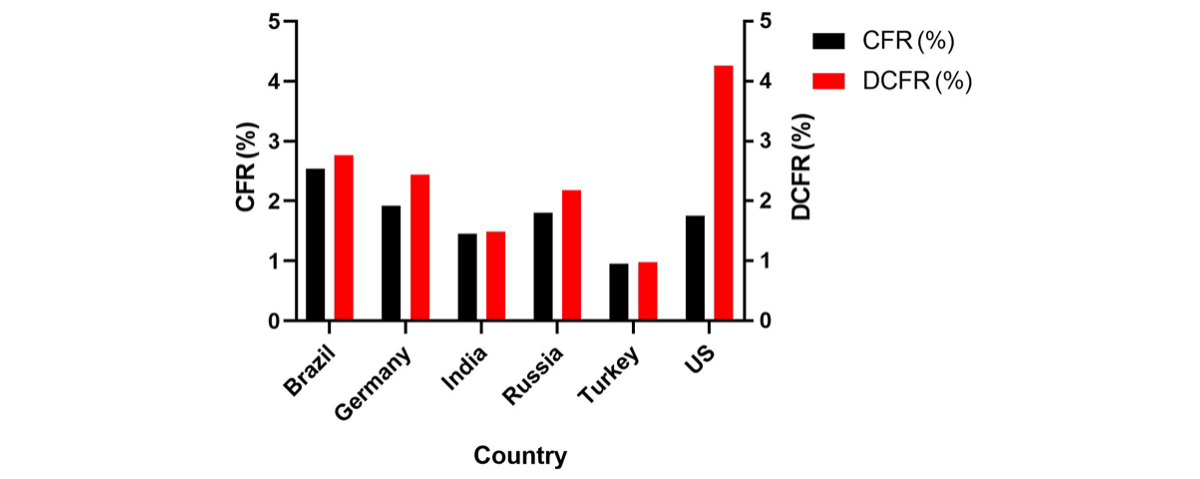
Epidemic situation in different countries. CFR: case fatality rate; tDCFR: total discharged case fatality rate.

By December 31, 2020, the total number of confirmed COVID-19 cases in the world reached 83,521,859; the total number of deaths reached 1,824,666; and the total number of recovered cases reached 47,032,627, with a CFR of 2.18% and tDCFR of 3.73%. We also analyzed COVID-19 data from the top 10 countries with the highest number of total confirmed cases. However, we were not able to calculate the DCFR for Spain, France, and the United Kingdom due to inaccurate data on the number of recovering patients. Among the other countries, the United States had the highest tDCFR (4.26%), while the tDCFR in the remaining countries ranged between 0.98% and 2.72% (Figure 4; Table 3).

**Table 3:** Worldwide COVID-19 statistics in 2020.

Country	Total confirmed cases, n	Total deaths, n	Total recovered cases, n	CFR^a^ (%), value (95% CI)	tDCFR^b^ (%), value (95% CI)
Brazil	7,675,973	194,949	6,848,844	2.54 (2.53-2.55)	2.77 (2.76-2.78)
Germany	1,760,520	33,791	1,350,708	1.92 (1.90-1.94)	2.44 (2.41-2.47)
India	10,266,674	148,738	9,860,280	1.45 (1.44-1.46)	1.49 (1.48-1.49)
Russia	3,127,347	56,271	2,527,722	1.80 (1.78-1.81)	2.18 (2.16-2.20)
Turkey	2,208,652	20,881	2,100,650	0.95 (0.93-0.96)	0.98 (0.97-1.00)
United States	20,099,363	352,078	7,919,313	1.75 (1.75-1.76)	4.26 (4.24-4.27)

^a^CFR: case fatality rate.

^b^tDCFR: total discharged case fatality rate.

## Discussion

### Reason for Proposing the Concept of the DCFR

The CFR is very crucial in the prediction of the epidemic trend because it reflects the degree of the danger that the epidemic poses [20]. The CFR is usually expressed in terms of the number of deaths per 100 treated patients [21]. However, in actual calculations, it is necessary to wait until the end of the epidemic when the total number of patients and total number of deaths can be counted, thereby providing accurate results. Therefore, it is difficult to use this formula to calculate the final CFR during the epidemic because the total number of patients and deaths is not conclusive. Moreover, during the epidemic period, the relevant departments sometimes replace the total death toll with the current number of deaths and replace the final confirmed number with the current confirmed number to get an approximate value of the CFR. However, this calculation method does not take into account the “number of patients cured and discharged.” Thus, it is not an appropriate measure of the medical level of hospitals. In addition, when the epidemic virus is unclear or the diagnosis ability is insufficient, the situation is more complicated and the number of patients is not accurate. Not only does the number of patients change daily, but also patients are divided into confirmed and suspected groups, and sometimes they will transform each other. Therefore, we propose a new method for calculating the CFR of ongoing infectious diseases, which is referred to as the DCFR (including the tDCFR and mDCFR). The DCFR refers to the ratio of the cumulative number of deaths to the sum of the cumulative number of deaths and the cumulative number of cured patients at a certain time point. Calculating the DCFR has several advantages. It can show the dynamic fatality rate in different regions in real time, it can evaluate the medical conditions in different regions, and it can scientifically guide and help arrange follow-up medical matters reasonably. In addition, after entering the “turning point,” the overall situation of the entire epidemic can be predicted in advance based on the development data of the epidemic at that time and with reference to the real-time dynamic fatality rate data.

### Comparing the CFR and DCFR With Real Data From Italy

Italy is one of the countries severely affected by the current COVID-19 pandemic [22,23], and thus, researchers have focused on assessing the evolution of the Italian epidemic. Italy was the first European country to face a massive outbreak of COVID-19 cases [24,25], where the epidemic started in the northern region in February 2020 and quickly spread to all regions of the country [26]. The Italian COVID-19 epidemic has been unique from several points of view, with more than 2,000,000 confirmed cases and a fatality rate estimated to be one of the highest in the world [27]. The CFR is an indicator that reflects the severity of the disease. It should have a relatively stable value under natural circumstances without other influencing factors. However, the CFR is affected by many factors in the early stage, where it first rises and then maintains a higher level for a period of time before falling. On the other hand, the CFR is stable in the later stage of a pandemic. Generally, the mortality rate in the early stage is relatively high due to an inadequate understanding of new infectious diseases in this stage, inadequate prevention and control measures, and unclear diagnosis. Therefore, the CFR cannot truly reflect the actual situation of the pandemic. In this study, we have used the trends of the DCFR and mDCFR to truly reflect the serious situation in the early stage of the COVID-19 epidemic in Italy. The obtained DCFR and mDCFR values showed that the COVID-19 fatality rate in Italy rose rapidly in February and reached a peak in March. Our results are consistent with the results of De Natale et al [22] who reported that COVID-19 was very serious in Italy in the early stage. After May, the fluctuation in the number of deaths gradually reduced the impact on the DCFR due to the large increase in the number of people discharged from the hospital, and finally, the DCFR remained stable. In a natural state without other influencing factors, the overall CFR should be relatively stable. In this study, we listed the numbers of confirmed cases, deaths, and recovered patients in each province in Italy, and used the numbers to calculate the CFR and tDCFR of each region. The results are presented on a map as shown in Figure 2. Some findings deserve attention from relevant departments of epidemic prevention and control. For example, the CFR and tDCFR values were both high in the Valle d'Aosta area despite the number of confirmed cases being low. In addition, the calculated DCFR was high in the Puglia area despite the CFR being very low. Therefore, the severity of COVID-19 in these areas cannot be ignored. This nonassociated result indicates that researchers should consider the CFR and tDCFR together with the determined number when assessing the severity of COVID-19. The DCFR may have a large error at the beginning of the pandemic, when the number of discharged cases is small, but the error will decrease as the number of discharged cases increases.

### Practical Applications of the DCFR

The role of public health institutions is to dynamically analyze the epidemic trend from the massive data after an outbreak, and provide government departments with both forward-looking and accurate professional prevention and control recommendations. Before the end of the epidemic, the epidemic management department and the general public are usually more concerned about the CFR of the epidemic, how many people are diagnosed with the infection, and how long the epidemic lasts. Only by clarifying these issues as early as possible can we accurately grasp the trend of the epidemic, and take targeted control measures to curb the rapid spread of the epidemic and avoid the generation of rumors and panic.

Prediction of the COVID-19 trend and control effects in the earlier stage is very important. This study has shown that the DCFR can be used as a predictor for the trend, and it can provide additional information for controlling diseases. Briefly, we proposed the value of the DCFR in describing emerging infectious diseases, divided the pandemic stages based on the mDCFR, and used the mDCFR to evaluate the control effect at different stages. We collected data from January 22 to December 31, and used joinpoint regression analysis to divide the data into 2 stages (Figure 3). Our DCFR results showed a gradually decreasing trend in the 2 stages (Table 2), which can be attributed to the characteristics of early cases [28], the improvement of diagnosis and treatment measures [3], government interventions, and the increase in the proportion of less dangerous viruses [18]. This suggests that it is reasonable to use the DCFR to predict the pandemic trend.

### Limitations

Although this study proposed the DCFR index first, some limitations should be addressed. We were not able to conduct some critical analyses due to unavailability of full data access in some countries. In our next study, we will try to find complete data from some key countries and use the data to calculate the indicators of the DCFR, such as age and gender. In addition, we will compare them with the findings in this study, classify the DCFR in detail in a larger database, and conduct long-term analysis.

### Conclusions

The DCFR can use ever-changing data to calculate a more accurate CFR, divide the pandemic stages of new infectious diseases, and analyze the dynamic trend. Our results suggest that the DCFR can be used as one of the pandemic control indicators. The results showed that the DCFR was high in the early stage of the COVID-19 outbreak in Italy, but it then decreased to a stable level. This suggests that other countries may also adopt the DCFR as one of the indicators of pandemic control. Furthermore, the DCFR may have potential application value in many emerging infectious diseases, such as Middle East respiratory syndrome and severe acute respiratory syndrome.

## Abbreviations

AMPC: average monthly percentage change
CFR: case fatality rate
CSSE: Center for Systems Science and Engineering
DCFR: discharged case fatality rate
mDCFR: monthly discharged case fatality rate
MPC: monthly percentage change
sDCFR: stage discharged case fatality rate
tDCFR: total discharged case fatality rate
